# Dynamic Response of the Skull with Sinuses under Blunt Frontal Impact: A Three-Dimensional Computational Study

**DOI:** 10.1155/2015/848079

**Published:** 2015-09-30

**Authors:** Xuewei Song, Botao Zhao, Cong Wang, Nan Wang

**Affiliations:** ^1^State Key Laboratory of Automotive Simulation and Control, Jilin University, Changchun 130025, China; ^2^First Bethune Hospital, Jilin University, Changchun 130021, China

## Abstract

The objective of this study is to analyze the biomechanical effects of sinuses in the skull on the facial impact response. Two models were built, where one had sinuses and the other had none. The models were verified using cadaver test data, including impacts to frontal bone, zygomatic bone, and maxillae. In the maxilla and zygoma impact, sinuses were found to have no significant effect on the global distribution of stress or stiffness of facial bones, and the influence was limited in local area. In forehead impact, the sinuses significantly affected the distribution of stress and strain in the skull due to its location in facial bones. The result shows that if the sinus is far away from the location of impact, its effect on the overall response of skull could be ignored. In addition, the distance between the region of interest and sinuses is another important parameter when studying the local effect of sinuses.

## 1. Introduction

Facial injuries caused by impact to facial areas are considered as a serious public health problem in both developed and developing countries [1–4]. Road traffic accidents are among the main causes of facial injuries [1, 5, 6] and can lead to disability and death. Facial injuries are often associated with dysfunction, facial bones fracture, and psychological problems [1]. Many investigations have been conducted using cadaver heads and physical head models to research the facial impact and facial bones injuries. In a study by Allsop et al. [7], the facial response of Hybrid III dummy and human cadaver was investigated by forehead, zygoma, and maxilla impact. The force-displacement curves of human cadaver were drawn. Nyquist et al. [8] conducted nasal impact experiments on eleven cadavers at Wayne State University. Fractures of the nasal bones were observed in all tests, and in some tests more extensive fractures were found, including one or more fractures of the maxilla, zygoma, and sphenoid bone. Cormier and Manoogian [9] conducted another cadaveric study to evaluate the response of cadaver subjects to blunt impacts to the frontal bone, nasal bone, and maxilla. The stiffness, fracture characteristics, material properties, and some structures of the face bone were investigated [7–16]. These experiments, together with development of computational techniques, have subsequently led to the development of numerical head models, especially finite element (FE) models, to allow more in-depth biomechanical studies [17]. Hardy and Marcal [18] and Nickell and Marcal [19] made the first attempts to build the FE model. However, only the skull was modeled [20]. After that, many detailed FE models with high biofidelity were built [17, 21–25]. With the finite element models, the distributions of the pressure, stress, and strain could be examined in the process of collision.

In recent years, the computational models of the head have been further developed in terms of scale and biofidelity. Zhang and Yang [23] developed a new version of the Wayne State University brain injury model to simulate the direct and indirect impacts, and the skull bone was modeled as a three-layer structure and assigned different materials, which was similar with the real skull bone. The cadaver tests of Allsop et al. [7] and Nyquist et al. [8] were simulated using this model. It can be seen that the model was of high biofidelity and could be used to predict the injury.

Although the biofidelity of the models has been improved, there are still some problems that should be paid attention to, such as the effect of sinuses on responses under facial impact. In anatomy, the sinus in bone is a cavity and the inner table of the sinus is cortical bone [26]. Most of the sinuses are found in the bones of the face and connecting with the nasal cavities. The response of the facial impact depends on the main structure of the face bone. However, there is little information in literature about the sinuses effect on the outcomes of facial impacts or the head injuries. A high-quality, extensively validated FE head model was developed by Mao et al. [17], and it was partially validated with 35 experimental cases, including facial impact. In this model, the sinuses are included, but they are developed from the CAD dataset and the geometries of the sinuses are simplified. In addition, Mao et al. did not further discuss the effect of sinus in modeling study. In some literature [27, 28], the idea of frontal sinuses as “shock absorbers” was raised and repeated and even used. As a result, the strain, stress, and other injury parameters would probably be influenced. Despite this, the idea of the sinus as protective structures remains completely untested [26]. It is also suggested that the bone with sinuses would be more deformable than the bones without sinus [28]. Even Roux considered that the areas of sinuses are not necessary for mechanical support [29].

The aim of the current study was to quantify the influence of sinuses on the dynamic response in 3D FE head models. In this paper, the skull model with sinuses was built based on the computed tomography (CT). The brain and other components were built according to the mesh of skull face. The skull biofidelity was ensured while the brain and cerebrospinal fluid (CSF) were simplified because the skull response in impact was the focus of our study. A new comparative model was constructed based on the model with sinuses by filling up the cavities of sinuses and deleting the inner cortical layer of sinuses. The models were validated against Allsop's facial impact experiments, respectively [7], including the forehead, zygoma, and maxilla impact. The comparison and analysis of these two models were conducted in order to investigate the influence of the sinuses on the facial impact.

## 2. Methods

### 2.1. Model Description

#### 2.1.1. The Geometry Model

In this present study, geometrical information of the human skull was obtained from axial images of 50th healthy Chinese male with a pixel size of 0.342 mm and slice thickness of 1.0 mm, collected from CT scan data. These medical images were imported into Mimics v10.01 (Materialise, Leuven, Belgium) for reconstruction of human skull without soft tissue. After the basic structure has been built, the geometries were imported into Geomagic Studio 12 (Geomagic, Morrisville, NC) for revising and building the surface. The geometry model was just the skull without other components, such as skin, brain, or muscle.

**Figure 1:**
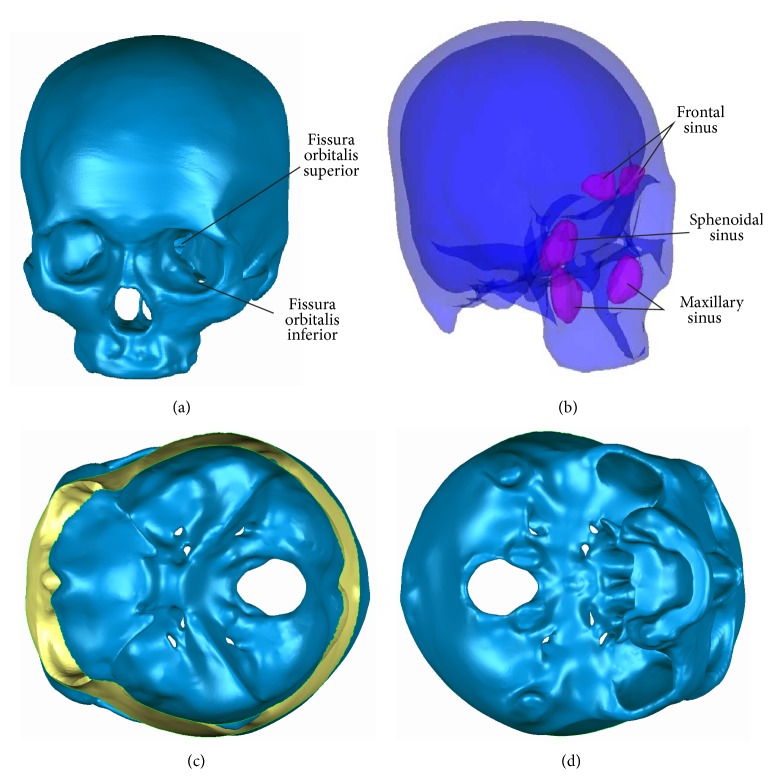
The geometry of the skull model. The anterior aspect (a), the location of sinuses (b), and the internal and external surface of the base of the skull geometry model (c, d).

During reconstructing the model, the skull geometry was built in detail. During dealing with the outer contours, some simplification is taken so the FE model could be constructed well. There were some parts still unclear just basing on the CT data so that the atlas of human anatomy was referred to. For the eye sockets, there are two fissures in each socket, the fissura orbitalis inferior and fissura orbitalis superior (Figure 1(a)). These structures were included in two models to keep the result accurate.

In order to study the sinuses effect, the accuracy of biofidelity was ensured, especially the sinuses. In this present paper, we concentrated on three kinds of sinus: frontal sinuses, maxillary sinuses, and sphenoid sinuses (Figure 1(b)). Efforts were made to model the interior and external shapes of the skull, such as the sulcus sinus petrosi superioris and the ala major (Figures 1(c) and 1(d)). Those would affect the transfer of the force during impacting.

#### 2.1.2. The FE Model

A semiautomatic meshing technique was employed in HyperMesh v11.0 (Altair HyperWorks, Troy, MI, USA). The total model consisted of a total of over 24900 nodes and 113600 elements, with a mass of 4.27 kg, including the scalp, skull, brain, and cerebral spinal fluid (CSF). The skull consisted of three layers, outer table, diploe, and inner table.

The architecture of the skull resembles a sandwich structure containing cancellous and cortical layers. The cancellous layer of the bone is generally thicker than the inner and outer tables of the skull. Thus, the inner and outer layers were defined as the shell elements with a thickness of 1 mm while the cancellous bone was modeled as solid elements which could present the varying thickness of the skull at different regions. The inner table of the sinuses was defined as shell elements with thickness of 1 mm, too.

The other components were modeled using tetra elements directly based on the elements of inner and outer face of the skull, including brain, CSF, and skin. In current study the structure of the brain was very simple and was not separated into cerebellum, corpus callosum, and other components, because the response of the facial bones was the focus of this study.

### 2.2. Material Properties

In the present study, the cancellous bone of facial and skull bones was defined as one component and the material was the same. The cancellous bone meshes were tetrahedral and the cortical bone meshes were trilateral.

An elastic-plastic material model was used for cortical and cancellous bone of the head. Element deletion available in the LS-DYNA material was introduced into this model to predict bony fracture [7]. The failure criterion of ultimate strain was used. This option removes any element with a strain that exceeds a preset ultimate strain magnitude in each time step. A Young's modulus of 4500 MPa was assumed for the cancellous bone, and the value used for cancellous bone was in the range found in the published literature [21]. A Young's modulus of 15 GPa was used for the cortical bone. Zhang and Yang [23] had cited that when using the Young's modulus of over 10 GPa, the stiffness is very high and one possible reason is that the human structure was not explicitly implemented. That was one of the reasons that we built the model with high fidelity. The material of face was the same as the material of cortical bone.

**Figure 2:**
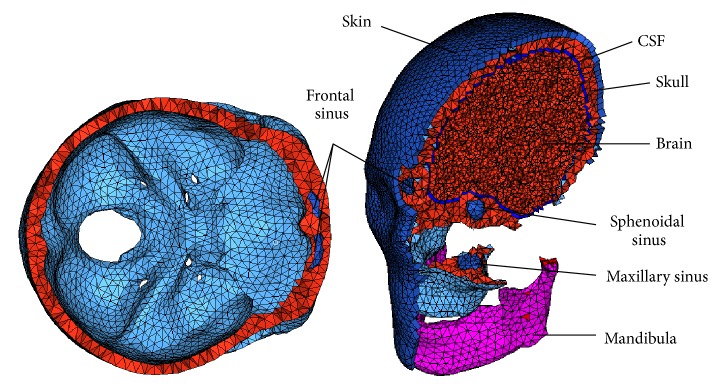
An overview of the baseline model with sinuses.

The material properties selected for the total head materials are listed (Table 1). There were two models in this study, and the model without sinuses (MWOS) was developed based on the model with sinuses (MWS) (Figure 2). They are all the same except the sinuses.

### 2.3. Experimental Data for Model Validation

Allsop et al. [7] conducted a series of facial impact experiments on fifteen cadavers and Hybrid III dummy to study the response of the skull and zygomatic and maxillary bones. The heads of cadavers, aged from 39 to 84, were fixed, facing upward. A 14.5 kg semicircular shaped aluminum rod impactor dropped from the height of 460 to 915 mm onto the frontal bone area and from 305 to 610 mm onto the zygomatic and maxillary regions. For the frontal bone impact, the longitudinal axis of the bar was set to impact the head approximately 20 mm above the supraorbital ridge. Zygomatic and maxillary impacts were performed at 10 mm below the suborbital ridge and 10 mm below the anterior nasal spine, respectively. The force-displacement curves and the cadaver facial stiffness curves were drawn. When compared with the cadaver results, the Hybrid III dummy face was several times stiffer in the midface region and should be redesigned. In current simulations, the impactor was the same with Allsop's experiments [7]. The velocities of impact were calculated from dropping height. When impacting the forehead and zygomatic and maxillary areas, the impactor was given initial velocities of 3.5 m/s, 2.7 m/s, and 2.7 m/s, respectively.

## 3. Result

### 3.1. Facial Impact Data Validation on Models

**Figure 3:**
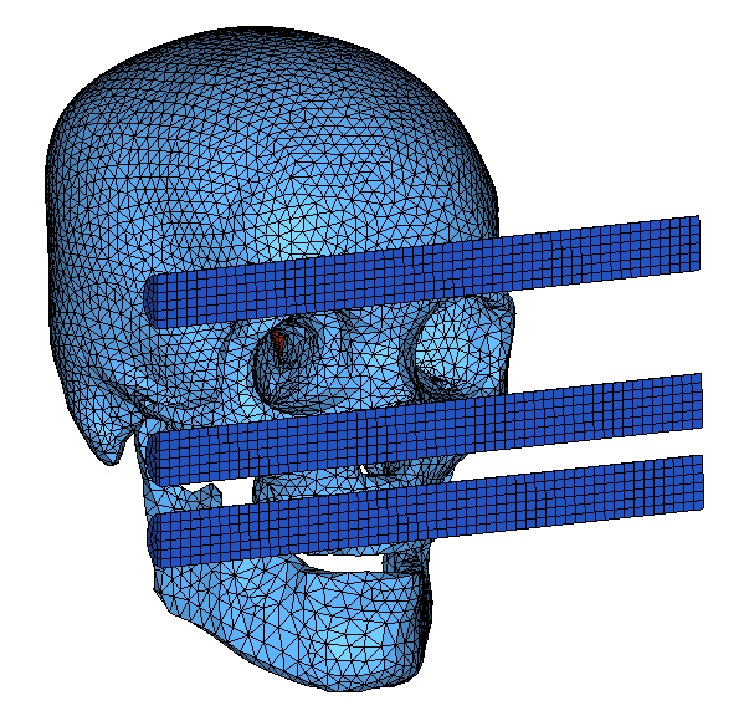
An oblique view of the forehead, zygoma, and maxilla impact location in simulations. The soft tissues of head are removed in this figure.

Figure 3 shows the simulation setup of the model for the facial impact tests conducted by Allsop et al. [7]. The velocity for each cadaver test was not reported and the average velocity calculated from dropping height was 3.5 m/s, 2.7 m/s, and 2.7 m/s, respectively. To validate the model due to forehead and zygomatic and maxillary impacts, the force-displacement response of the model was calculated.

**Figure 4:**
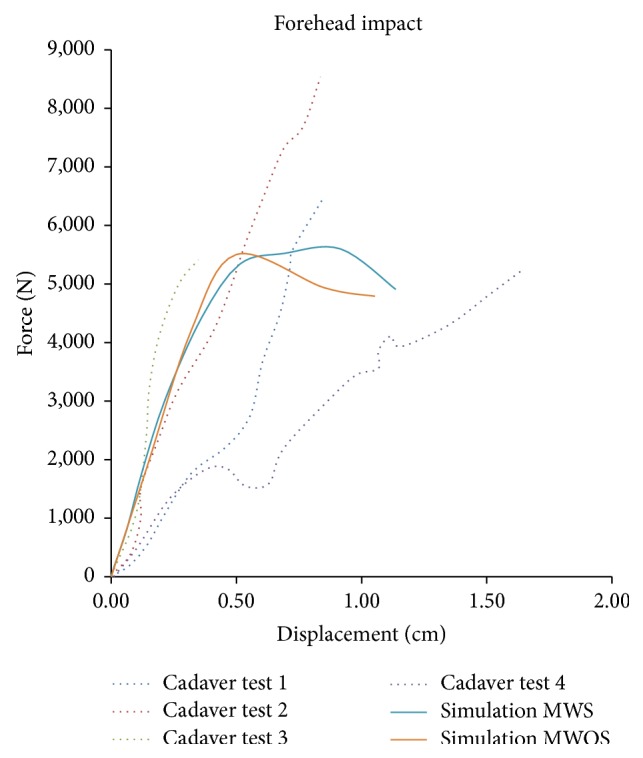
Comparison of force-displacement for the forehead impact between experimental measurements (Allsop et al.) and model predictions.

Figure 4 shows the results of a forehead impact simulation plotted against cadaver test data by Allsop et al. [7]. As depicted in the figure, the peak force and stiffness before fracture fell well within the range of the test results, but the fracture was bigger than the hairline fracture observed in Allsop's tests [7]. The contact force in simulation reduced after fracture, which was different with cadaver tests. The MWS and MWOS were consistent before the displacement reached 0.5 cm, and it was after that point that the fracture happened. However, when the fracture occurred (displacement over 0.5 cm), the force-displacement histories were different between two models. The contact force of MWOS decreased more quickly while that of MWS was stable for a while.

**Figure 5:**
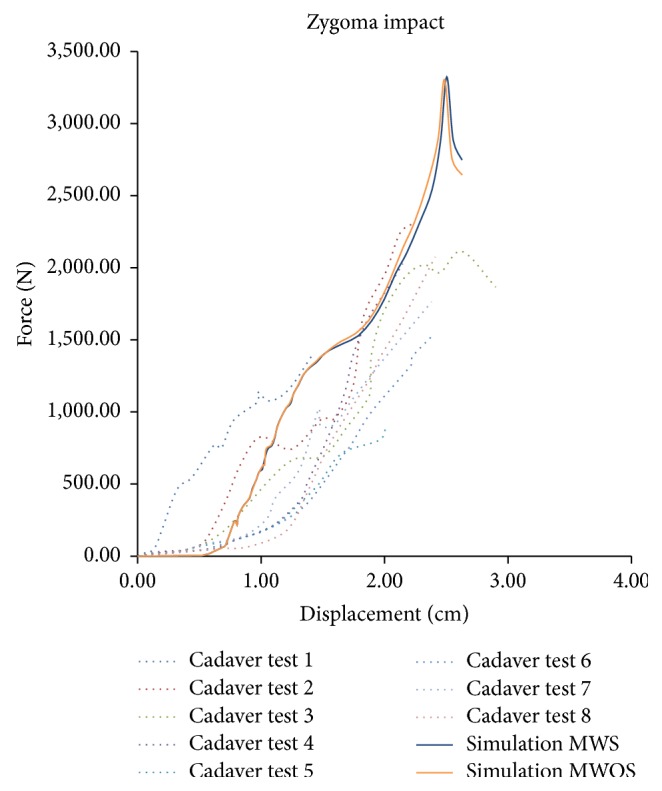
Comparison of force-displacement for the zygoma impact between experimental measurements (Allsop et al.) and model predictions.

Figure 5 is a comparison of force-displacement history for zygoma impact. Model predictions agreed well with the test data and the stiffness for each model was acceptable. Also there was no obvious distinction between these two models. However, the stiffness had an increase after 0.7 cm. Also, the peak value was higher than the test, which is most likely duo to the material and structure of nasal bone. The MWS and MWOS peak values are 3320 N and 3300 N, respectively. The peak force reduction in the model with sinuses compared to the model without sinuses was 0.6%. Since fracture patterns were not reported in Allsop's study [7], the fractures in this simulation were not investigated.

**Figure 6:**
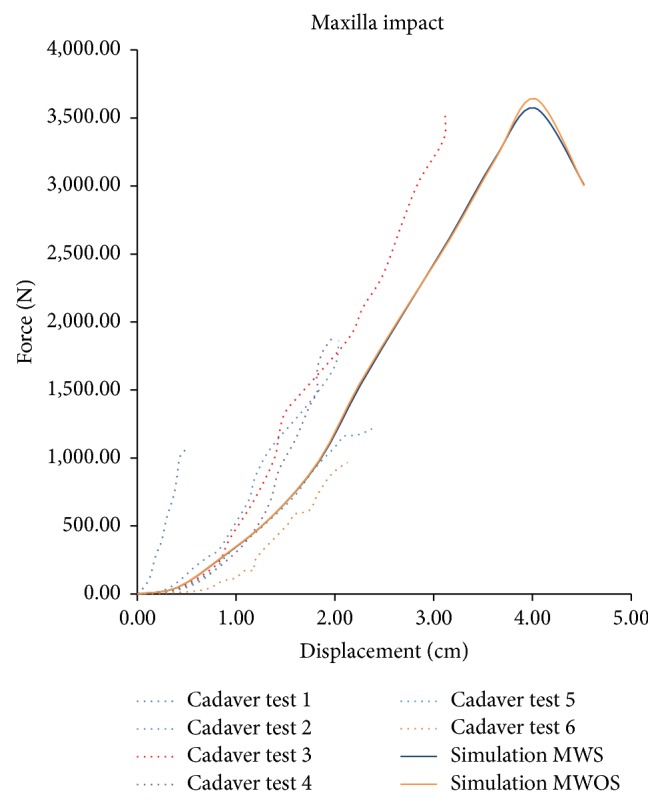
Comparison of force-displacement for the maxilla impact between experimental measurements (Allsop et al.) and model predictions.

Figure 6 shows a comparison of force versus displacement for maxilla bone impact. These two models were nearly the same. The stiffness of the head model matched the tests, but the peak contact force was much higher than the average force of cadaver tests. The fracture happened when the force reached 3500 N. The skin part was reflected by a relatively flat portion for about five millimeters followed by a change in slope indicating increasing stiffness.

### 3.2. Comparison of Maximum Principal Stress

**Figure 7:**
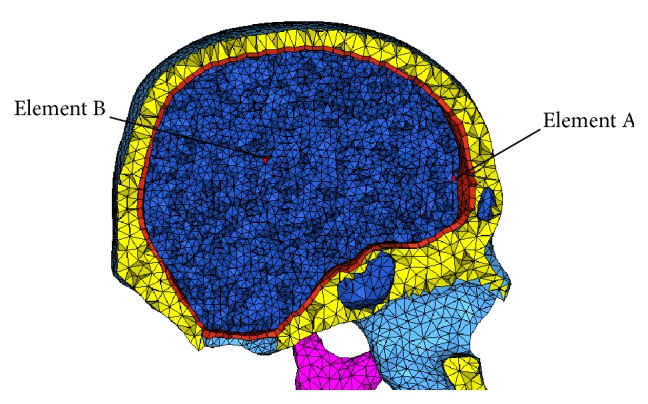
The location of elements A and B.

Besides the force-displacement histories, the stress in the same direction during impacting was compared between these two models. In order to show the influence of the sinuses to the total head, two elements in each impact were chosen. Element A is near the sinuses and located in the front of the brain, while element B is far from sinuses and located in the center of the brain. The location of elements was showed in Figure 7 and the comparison of peak value was listed in Table 2.

**Figure 8:**
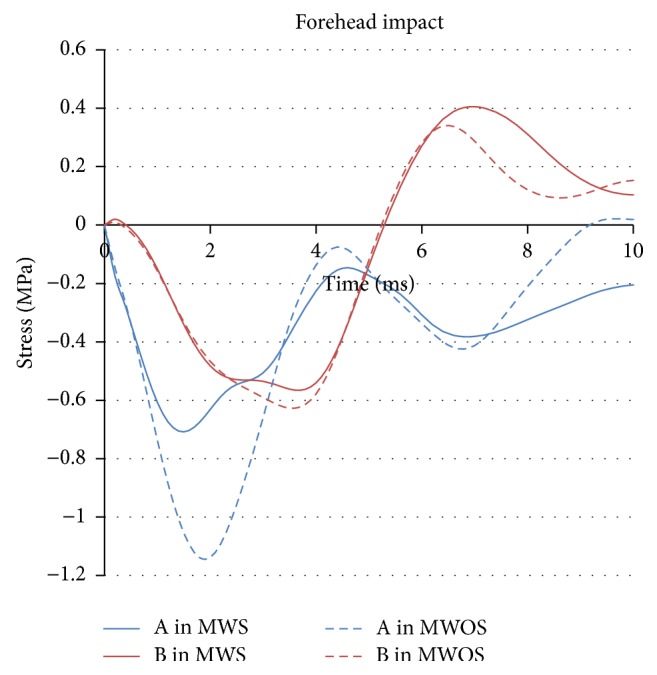
The stress-time history of elements in two models in forehead impact.

The history of maximum principal stress of two elements in different model for forehead impact is shown in Figure 8. The curvilinear trend of two elements is similar. However, the peak of negative stress of element A in MWOS reached −1.14 MPa, while in MWS the peak value was only −0.70 MPa, with a magnitude reduction of 38%. In the middle of the brain, the peak value of negative stress was about −0.6 MPa, no matter MWS or MWOS. It is obvious that the sinuses made a difference, and the influence was great in the front of the brain. In the middle of the brain, the difference was reduced, and the peak value in MWS was lower than that in MWOS only by a reduction of 10%. The positive stress only appeared in the middle of the brain, and the MWS experienced 17% increment, compared with MWOS. Therefore, the results of MWS and MWOS were palpably different because of the sinuses in forehead impact.

**Figure 9:**
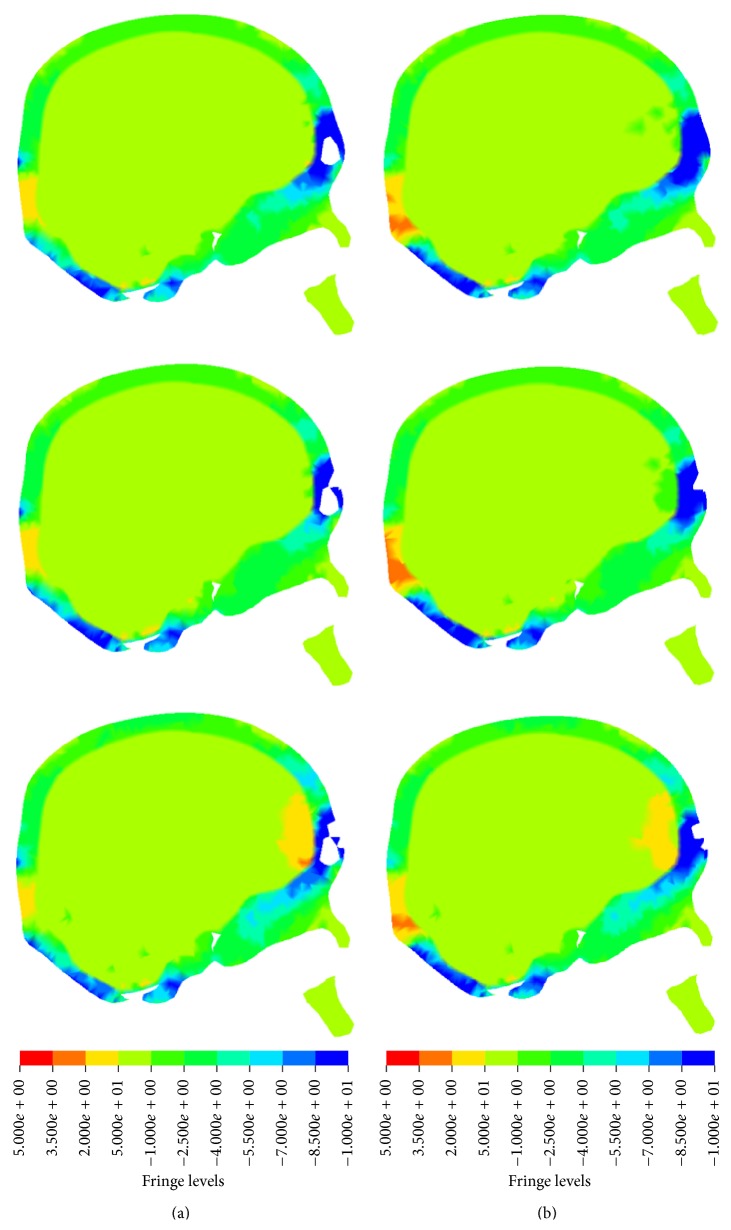
The stress distribution in two models at the time of 2 ms, 2.2 ms, and 2.4 ms in forehead impact. Column (a) is from MWS and (b) is from MWOS.

The contours of stress in forehead impact were compared between two models (Figure 9). There was some little area with high stress in brain of MWOS at the time of 2 ms. At the time of 2.2 ms, there was a local area with high stress behind the impact location in MWOS, while there was not in MWS. At the same time, the fracture happened in two models. The stress distribution came to be similar quickly after the fracture, which was agreed with the stress-time history.

**Figure 10:**
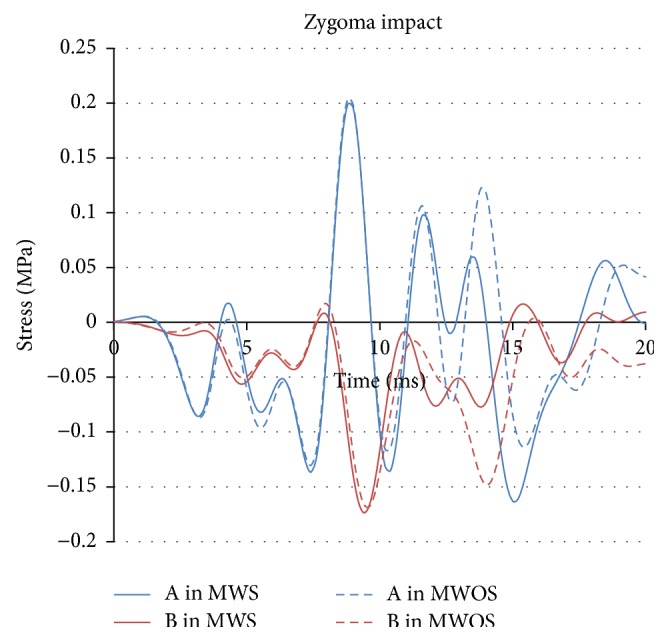
The stress-time history of elements in two models in zygoma impact.

**Figure 11:**
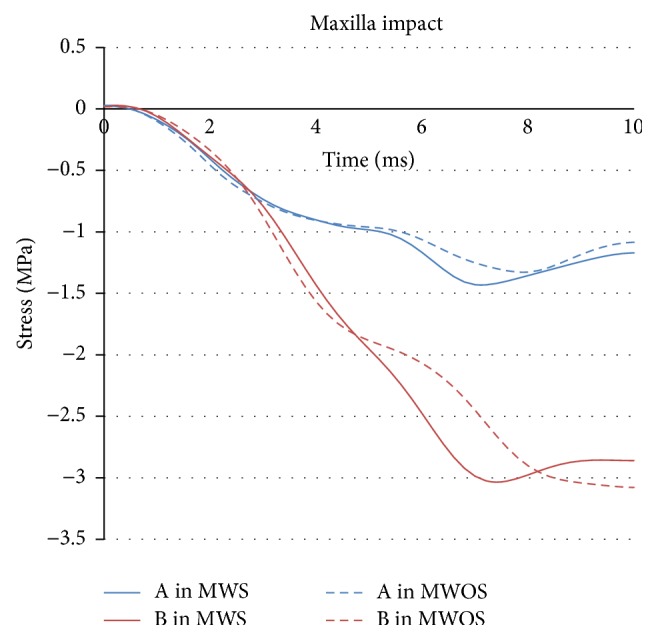
The stress-time history of elements in two models in maxilla impact.

In zygoma and maxilla impacts, the peaks of the stress (Figures 10 and 11) were similar between MWS and MWOS, no matter in the middle or the front of the brain. It could be seen that the curves were nearly the same in entire process. It is probably because the sinuses were far from the impacting position, unlike the forehead impact. There was only negative stress in maxilla impact. In the frontal of the brain, the stress of MWS increased by 7%. In the middle brain, the stress was more than −3.00 MPa in two models, but the reduction was only 1%. In the zygoma impact, the stress was very similar, and the reduction was below 7%. Also, the difference was magnified by the percentage due to the small base. The distribution was nearly the same in the contours of stress, too. Therefore, in zygoma and maxilla impacts, the sinuses did not make obvious difference in terms of intracranial stress. In addition, the conclusion could be drawn that the reduction in front of the brain was bigger than that in the middle. The details of the stress were listed in Table 2.

### 3.3. Comparison of Effective Strain

**Figure 12:**
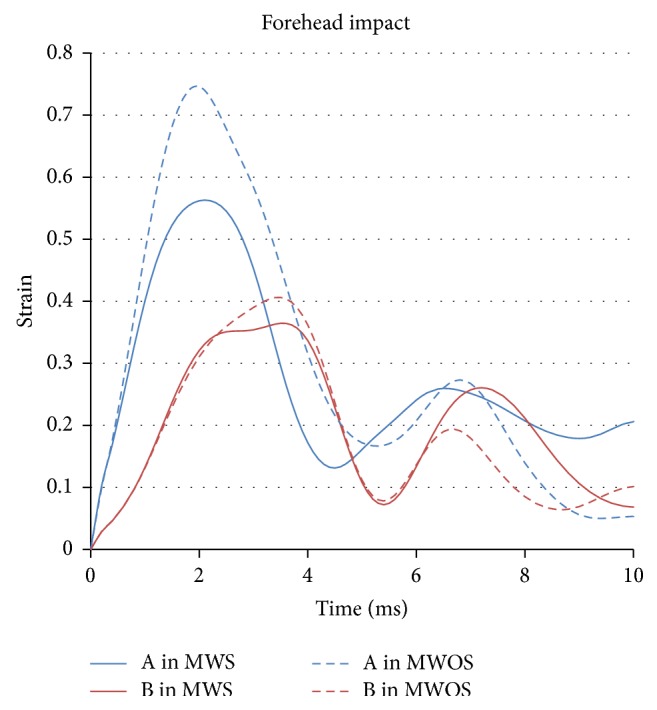
The strain-time history of elements in two models in forehead impact.

Besides the stress, the middle surface effective strain of element in two models was compared, too (Table 3).  Figure 10 is the stress-time history in forehead impact. The strain of element A in two models reached the peak of 0.75 and 0.56 at the time of 2 ms when the fracture just happened. The strain of A in MWOS was higher than that in MWS by 34%. In element B, the difference between two models was much smaller, and the strain in MWOS was a little higher than that in MWS by 11% (Figure 12). From the aspect of strain, the effect of the sinuses in strain was similar to that in stress in forehead impact.

**Figure 13:**
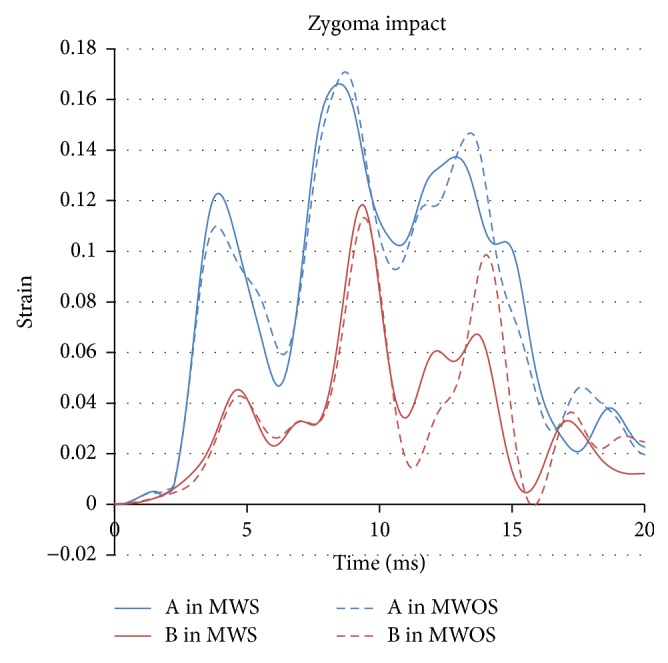
The strain-time history of elements in two models in zygoma impact.

**Figure 14:**
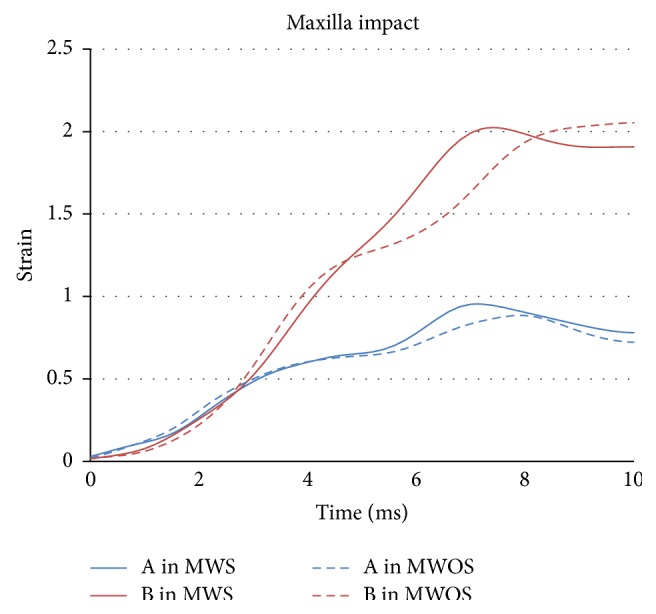
The strain-time history of elements in two models in maxilla impact.

In zygoma and maxilla impacts (Figures 13 and 14), the influence of sinuses in strain agreed well with that in stress, too. In particular, in maxilla impact, the trend of strain was similar with that of stress. The peak strain of A reached 0.8 in MWOS, while it reached 0.9 in MWS, with an increase of 11%. The peak strain in B was similar by a reduction of 2%. In zygoma impact, the difference between two models was very small and the difference in percentage was 4% and 3%, respectively.

## 4. Discussion

The two models were validated against three cases of impact by Allsop et al. [7]. In general, the models were able to predict the response of facial impact. The force-displacement histories showed that the stiffness of the model face was similar to cadaver tests. However, in forehead impact, the fracture of simulation was bigger than that of cadaver tests, and it properly was the reason why the contact force after fracture in simulation was different with that in cadaver tests.

In zygoma impact, the change of stiffness after 0.7 cm is probably due to the structure of the nasal bone. Cormier and Manoogian [9] also pointed out that the toe region of the response varied significantly because of the variation in nasal geometry. The length of the nasal bone in impacting direction would influence the depth at which the impactor would interact with the nasal bone after initial contact with the nose. The peak forces are higher than cadaver tests. The higher peak force was likely due to the thickness and the material of the facial bones. The material of the facial bones and skull was the same in this paper. We had changed the facial material into another soft one, and the elastic modulus was 5000 MPa. However, the stiffness is out of acceptable range and large deformation occurred at the facial bones during forehead, maxilla, and zygoma impacts, but the peak force in maxilla impact reduced to 2250 N. It showed that the material of maxilla was softer than the material of frontal bones and the parameter of element failure criterion probably should be changed. We will solve the problem in next hexahedron mesh model.

The current study indicates that the influence of the sinuses in the forehead impact is more significant, while in the zygoma and maxilla impacts the influence is minimal.

In forehead impact, the stiffness of two models was the same before the fracture; the force-displacement histories were the same, too. The sinus should be considered as one entity and transmit the force straightly at this period. However, it made the distribution different. Once the fracture happened, or the sinuses were broken, the situation differed more. At the beginning, the structure of sinuses was completed and the force could transmit in similar way. While the sinus was broken down or the fracture happened, the structure was changed and transmission of force changed, too. However, the cavity cannot transmit or absorb much energy and then more deformation appeared. This is the reason why the contact force-time history in MWS did not reduce immediately but the stress was smaller. The conclusion can be drawn from the stress-time and strain time history in forehead impact, too. The stress and train reached the peak at the same time which was about 2 ms. However, the stress and strain of element A in MWOS were higher by about 35% than that in MWS, while the difference in element B was very little. It was also obvious in the contours of stress that sinuses made the difference in local area, but the stress was similar in other areas in two models. The results showed that the sinuses make a significant difference in local area, no matter in stress or strain. The influence was sharply decreased in the location far from the sinus.

In zygoma and maxilla impacts, global reactions of two models were the same, and the reason was that the sinuses were too far from the impact location. In other words, the sinuses did not make the global effect, except in the local area around the sinuses. In addition, the effect was limited and was like that in forehead impact. When the impactor hit the bone, the force could transmit without the effect of sinuses. It was different with forehead impact, in which the structure was broken at the beginning of the fracture and, as a result, the force and energy were changed at once. In zygoma and maxilla impacts, the sinuses did not change the global situation, while in forehead impact the sinus did. The sinuses did not influence the stiffness of the facial bones or the global stress or strain distribution, and the weak effect was limited in local area around the sinuses.

It was obvious that the difference in element A is bigger than that in element B in all impacts, which means the effect of sinuses diminished following the distance increasing. If the sinus worked, the difference in the area around it was most apparent. Based on the comparison of two models, the conclusion can be drawn that the distance between the sinuses and the position of impact was the key whether the sinuses would make a significant effect on the response of facial impact. When the sinuses are far from the location of impact, the global influence of sinuses can be ignored, or otherwise sinuses would make some difference.

## 5. Conclusion and Limitation

In summary, a new 3D finite element model with sinuses and anatomy structure has been developed. The model has been validated against tests of facial impact. The result correlated well with forehead impact, zygomatic impact, and maxillary impact.

More importantly, the effect of sinuses was investigated by comparing two models. The results showed that the sinuses had no global effect in zygomatic impact and maxillary impact. In forehead impact, the global effect of the sinus was limited. In all impacts, the sinus would make a difference in local area. However, the difference is significant in forehead impact, while the differences are smaller in zygoma and maxilla impacts.

When the impact location is near the sinus, such as forehead impact in this paper, the sinuses would make a global influence and a significant influence in local area. If the location of impact is far from the sinuses, the global influence is very small and can be ignored, and the local effect depends on the distance between the sinus and location of impact.

However, the sinus ethmoidales was not far from the impact location. It is a porous structure and the number of cellulases in it differs from person to person. In common, there were 3 to 18 cellulases in one sinus ethmoidales. In current study, the sinus ethmoidales was not included, which probably would affect the fracture position. A more accurate FE model may help to study the issue in more detail.

## Figures and Tables

**Table 1:** Mechanical properties of materials.

Component	Property	Density (kg/m^3^)	*E* (MPa)	*K* (MPa)	Poisson's ratio	*G*_0_ (kPa)	*G*_*∞*_ (kPa)	*β* (s^−1^)

CSF	Viscoelastic	1040	—	2190	—	0.5	0.1	80
Brain	Viscoelastic	1060	—	2190	—	6	1.2	80
Skin	Elastic	1100	16.7	—	0.42	—	—	—
Cortical bone (shell)	Elastic plasticity	2100	15000	—	0.25	—	—	—
Cancellous bone (solid)	Elastic plasticity	1000	4500	—	0.30	—	—	—
Aluminum	Rigid	2700	70000	—	0.33	—	—	—

**Table 2:** The comparison of stress (MPa) between models.

		Element A		Element B	
		Upper peaks (MPa)	Lower peaks (MPa)	Upper peaks (MPa)	Lower peaks (MPa)
Frontal	MWOS	—	−1.14	0.34	−0.63
	MWS	—	−0.70	0.40	−0.57
	Change	—	−38%	+17%	−10%

Zygoma	MWOS	0.20	−0.15	—	−0.17
	MWS	0.20	−0.16	—	−0.17
	Change	0	+7%	—	0

Maxilla	MWOS	—	−1.33	—	−3.08
	MWS	—	−1.43	—	−3.04
	Change	—	+7%	—	−1%

**Table 3:** The comparison of middle surface effective strain between models.

		Element A	Element B
Frontal	MWOS	0.75	0.40
	MWS	0.56	0.36
	Change	−34%	−11%

Zygoma	MWOS	0.173	0.116
	MWS	0.167	0.119
	Change	−4%	+3%

Maxilla	MWOS	0.8	2.10
	MWS	0.9	2.05
	Change	+11%	−2%
